# Plastome sequences help to improve the systematic position of trinerved *Lindera* species in the family Lauraceae

**DOI:** 10.7717/peerj.7662

**Published:** 2019-10-07

**Authors:** Xiangyu Tian, Junwei Ye, Yu Song

**Affiliations:** 1 Ministry of Education Key Laboratory for Biodiversity Science and Ecological Engineering, College of Life Sciences, Beijing Normal University, Beijing, China; 2 Germplasm Bank of Wild Species in Southwest China, Kunming Institute of Botany, Chinese Academy of Sciences, Kunming, Yunnan, China; 3 Center for Integrative Conservation, Xishuangbanna Tropical Botanical Garden, Chinese Academy of Sciences, Xishuangbanna, Yunnan, China; 4 Southeast Asia Biodiversity Research Institute, Chinese Academy of Sciences, Yezin, Nay Pyi Taw, Myanmar

**Keywords:** Lauraceae, Complete plastid genome, *Lindera*, Phylogenetic relationship, Comparative genomics

## Abstract

*Lindera* is a genus (c. 100 spp.) of trees belonging to the “core Laureae” group in the family Lauraceae. It is often confused with *Litsea*, and the systematics of the genus is unclear. Here, total 10 complete plastomes from nine trinerved *Lindera* species and another species *Lindera obtusiloba* (sect. *Palminerviae* Meissn.) were sequenced. Nine highly variable regions, *trnH*-GUG/*psbA*, *psbM*/*trnD*-GUC, *petA*/*psbL*, *ndhF*, *trnL*-UAG/*ndhD*, and *ycf1*, were identified among the 10 *Lindera* species. In addition, a total of 1,836 mutation events including six micro-inversions, 156 indels, and 1,674 substitutions, were also summarized. Comparing our sequences with other available plastomes in the “core Laureae,” we put forward that six hypervariable loci, *trnH*-GUG/*psbA*, *ndhF*, *ndhF*/*rpl32*, *trl32/trnL-*UAG, *ndhD*, and *ycf1*, could potentially be used as plastid barcode candidates for species identification. Further phylogenetic analyses were conducted using 49 complete Lauraceae plastomes. The results supported a close relationship among trinerved *Lindera* species and suggested an improved trinerved group comprising species of trinerved *Lindera* species and *Iteadaphne caudate*.

## Introduction

The “core Lauraceae” group, originally defined by Rohwer & Rudolph (2005), consists of the Laureae-Cinnamomeae group and Persea group. Accumulating evidence shows a number of problems in the systematics of the intra- or inter-genus. The genus *Lindera* Thunb., a member of the “core Laureae” is widely distributed in the tropical to temperate regions of Asia, North America, and even Australia (Hyland, 1989), with 38 of the 100 known species in China alone (Li et al., 2008b). It has a close relationship with *Litsea*, *Neolitsea*, *Laurus*, *Actinodaphne*, etc., as shown in Chanderbali, van der Werff & Renner (2001). It appears to be polyphyletic according to its current constitution, despite the precise generic boundaries and species delineations based on morphological characters being currently unclear. *Lindera umbellata* Thunb., a deciduous shrubby tree with pinnately compound leaves, is the type species. Following Hooker’s (1890) criteria and Li’s (1985) concept, Tsui (1987b) divided *Lindera* into eight sections using characteristics such as being evergreen or deciduous, having penninerved or trinerved leaves, well-developed or non-developing terminal buds, non-enlarged or enlarged perianth tubes, and normal or shortened brachyblasts. However, these divisions were not explicitly phylogenetic (Chanderbali, van der Werff & Renner, 2001; Li et al., 2008a; Rohwer, 2000).

Using the plastid marker *matK*, the first molecular study on *Lindera* suggested that *Lindera benzoin* (L.) Blume together with other “core Laureae” represents a weakly supported paraphyletic group (Rohwer, 2000). Another phylogenetic study comparing different combinations of plastid sequences (*trnL-trnF*, *psbA-trnH*, *trnT-trnL*, and *rpl16*) and nuclear barcoding markers (26S rDNA and ITS rDNA), showed a well-supported “core Laureae” group comprising three *Lindera* species, *Lindera benzoin*, *Lindera erythrocarpa* Makino, and *Lindera umbellata* Thunb., as well as other species in their ITS analysis (Chanderbali, van der Werff & Renner, 2001). However, in their *trnL-trnF* + *psbA-trnH* analysis, the “core Laureae” group had no support from the paraphyletic relationships among these species. Although the reports using ITS or the single-copy nuclear marker *rpb2* (including different plastid markers) also confirmed that the “core Laureae” group and *Lindera* were polyphyletic (Fijridiyanto & Murakami, 2009; Li et al., 2008a; Liu et al., 2017; Nie, Wen & Sun, 2007), the question still remained: were there any useful sequences for the phylogenetic classification of the “core Laureae” group in plastid genomes?

Trinerved and pinninerved-leaf *Lindera* species were merged into a single genus: 13 species of *Lindera* with trinerved leaves in sect. *Daphnidium* (Nees) Benth., and one species in sect. *Uniumbellae* H.P. Tsui and Subg. *Iteodaphne* (Bl.) Kosterm (Li et al., 2008b). The complex relationships between the 34 Lauraceae taxa had been well resolved based on plastome sequence data, and included five *Lindera* species: *Lindera aggregata*, *Lindera obtusiloba* Blume, *Lindera megaphylla*, *Lindera communis* Hemsl. and *Lindera glauca* (Siebold & Zucc.) Blume (Song et al., 2017b). In this study, one of the trinerved leaf species, *Lindera aggregate*, was found to be more closely related to the *Actinodaphne*-*Neolitsea* clade than to other species of *Lindera* and *Litsea*, similar to trees inferred from *rpb2* (Fijridiyanto & Murakami, 2009; Song et al., 2017b). Most recently, another phylogenomic analysis using four complete plastomes of *Lindera latifolia* Hook.f., *Lindera robusta* (C.K. Allen) H.P. Cui, *Lindera metcalfiana* C.K. Allen and *Lindera nacusua* (D. Don) Merr. confirmed that nine *Lindera* species grouped into two strongly supported clades. One clade consisting of five species of *Lindera* formed a sister group to the second, which included *Laurus nobilis*, *Litsea glutinosa*, and four other species of *Lindera* (Zhao et al., 2018). However, the scarcity of complete plastid genomes of trinerved species in *Lindera* seriously impedes species identification and phylogenetic relationship determination. Thus, the phylogenetic relationships and genome information of trinerved *Lindera* truly needs further study.

Typical plastid genomes in most angiosperm species, which have maternal inheritance and haploidy, show high resolution in land plants with complex relationships (Barrett et al., 2016; Daniell et al., 2016; Ma et al., 2014; Wolfe & Randle, 2004; Zhang et al., 2017). Until now, over 43 (38 species, 19 genera) Lauraceae accessions with complete plastid genomes have been assembled and submitted to GenBank at the National Center for Biotechnology Information (data: April 11, 2018). The lengths of these complete genomes range from 114,622 bp (*Cassytha filiformis* Meisn, accession: MF939338) to 158,530 bp (*Beilschmiedia tungfangensis* S.K. Lee & L.F. Lau, accession: MF939348) (Song et al., 2017b, 2019). Among these plastids, eight intergenic spacers (*accD-psaI*, *ndhC-trnV*, *ndhF-rpl32*, *psbM-trnD*, *rpl32-trnL*, *rpl14-rps8*, *rps3-rps19*, and *rps16-trnQ*), three gene segments (*rbcL*, *ycf1*, and *ycf2*), and three introns (*clpP*, *ndhA*, and *trnG*-UCC) showed promising levels of variation for application in DNA-barcoding or intrageneric studies in Lauraceae (Hinsinger & Strijk, 2017; Song et al., 2015, 2016, 2017b, 2018; Zhao et al., 2018).

The genera *Laurus*, *Lindera*, and *Litsea* are closely related—they all share similar morphological traits such as being dioecious, having umbellate inflorescences subtended by large involucral bracts, having all introrse anthers, and having two stipitate glands at the third whorl (Li et al., 2004; Li & Christophel, 2000). Divisions based on only one characteristic may result in “twin species” (Ng, 2005), such as *Lindera*/*Litsea*, *Parasassafras* D.G. Long/*Sinosassafras* H.W. Li, *Dodecadenia* Nees/*Iteadaphne* Blume, differing only in 2- or 4-locules. Curiously, phylogenetic analysis of “core Laureae” based on single copy nuclear *rpb2* gene sequences and complete plastid genomes have found that *Lindera aggregata* was found to be more closely related to the *Actinodaphne*-*Neolitsea* group than to other species of *Lindera* and *Litsea* (Fijridiyanto & Murakami, 2009; Song et al., 2017b). This was unanticipated because of the different numbers of celled anthers between *Lindera* and the *Actinodaphne*-*Neolitsea* group. Additionally, trinerved leaf *Lindera* species when merged with pinninerved ones into one genus would be morphologically homoplasious. Therefore, the study of the phylogenetic relationships and genome information of trinerved *Lindera* still needs improvement.

In this study, we further sequenced nine samples of seven trinerved *Lindera* species, plus one from *Lindera obtusiloba*, using next-generation sequencing technology. In our assessment of plastid genomes of the nine morphologically similar trinerved *Lindera* species with one trinerved or pentanerved species, we will address the following objectives: (1) investigate the size range of complete plastid genomes; (2) examine the type of mutation events and variation regions; and (3) analyze the phylogenetic relationships with other “core Laureae” species in the family Lauraceae. The results will provide further information for *Lindera* species identification and taxonomic revision.

## Materials and Methods

### Material preparation and DNA sequencing

Dry leaves of all *Lindera* species were deposited in the Herbarium of the Xishuangbanna Tropical Botanical Garden (HITBC), Chinese Academy of Sciences (CAS) (Table 1). High-quality genomic DNA was isolated from ca. 6 cm^2^ sections of dry leaf using an improved CTAB method (Yang, Li & Li, 2014). Total DNA (0.5 μg) was checked on the Agilent BioAnalyzer 2100. Libraries with short-inserts of 500 bp in length were generated with an Illumina Paired-End DNA library Kit according to the manufacturer’s protocol (Illumina, San Diego, CA, USA). Genomic DNA samples from each species were indexed with tags and pooled together in one lane of Illumina Genome Analyzer Hiseq 2000 for sequencing by Beijing Genomics Institute (Shenzhen, China).

**Table 1:** Comparison of complete plastid genome of 10 *Lindera* species.

Taxa	*L. aggregata*	*L. chunii*	*L. limprichtii*	*L. pulcherrima*	*L. obtusiloba*	*L. supracostata*	*L. thomsonii* BOP	*L. thomsonii* SY	*L. thomsonii* var. vernayana	*L. fragrans*
Voucher	SY01432	SY34518	SY34167	SY34019	SY35538	SY34832	SY34836	SY34818	SY35026	SY34828
Geographic origin	Yunnan, China	Guangdong, China	Gansu, China	Guizhou, China	Shanxi, China	Yunnan, China	Yunnan, China	Yunnan, China	Yunnan, China	Yunnan, China
Coverage	180×	174×	137×	136×	168×	105×	135×	146×	567×	123×
Total length (bp)	152,717	152,734	152,696	152,693	152,717	152,662	152,673	152,649	152,626	152,644
LSC (bp)	93,733	93,716	93,748	93,726	93,637	93,698	93,711	93,702	93,669	93,681
SSC (bp)	18,822	18,838	18,816	18,837	18,912	18,824	18,830	18,815	18,825	18,823
IR (bp)	20,081	20,090	20,066	20,065	20,084	20,070	20,066	20,066	20,066	20,070
GC content (%)	39.1	39.2	39.1	39.1	39.2	39.2	39.2	39.1	39.2	39.2
Total number of genes (unique)	127 (113)	127 (113)	127 (113)	127 (113)	127 (113)	127 (113)	127 (113)	127 (113)	127 (113)	127 (113)
Protein encoding	83	83	83	83	83	83	83	83	83	83
tRNA	36	36	36	36	36	36	36	36	36	36
rRNA	8	8	8	8	8	8	8	8	8	8

### Genome assembly and annotation

The low-quality score paired raw reads were filtered and trimmed, and de novo assembled to form contigs using GetOrganelle software (Jin et al., 2018). The assembled contigs were searched against the referenced plastid genome sequence of *Laurus nobilis* L. (accession: KY085912) using a BLASTn (ncbi-blast-2.6.0) (Hinsinger & Strijk, 2017). The high similarity contigs were created using a consensus mapping sequence in Geneious R11 (Kearse et al., 2012). The generated consensus sequence was used as a reference to map high-quality trimmed reads to correct ambiguous nucleotides in Geneious R11 and to extract a new consensus sequence as the final circular plastid genomic sequence. The plastid genomes were annotated using a web-based annotation program GeSeq (Tillich et al., 2017) with an uploaded reference of *Laurus nobilis* L. Protein, rRNA, and tRNA were annotated with BLASTn, CDS, and rRNA using HMMER search (Finn, Clements & Eddy, 2011). tRNA was checked with tRNAscan-SE v1.3.1 (Lowe & Eddy, 1997), which is part of the GeSeq programs. The output GenBank files were inspected and edited manually, and plastid genome circle maps were drawn using OGDraw v1.2 (Lohse et al., 2013).

### Whole genome comparison and divergence hotspot identification

Repetitive genomic sequences were identified using the REPuter web-service program (Kurtz et al., 2001) to distinguish forward, reverse, complemented, and palindromic repeats, with a 30 bp minimal repeat size and more than 90% sequence identity (hamming distance equal to 3). Simple sequence repeats (SSRs) were detected using MISA-web (http://webblast.ipk-gatersleben.de/misa/) with the following parameters: 10, five, four repeat units for mono-, di-, tri-nucleotides and three repeat units for tetra-, penta-, hexa-, 7-, 8-, 9-, 10-nucleotides (Beier et al., 2017). Indel events were counted after aligning the nine trinerved *Lindera* species plastid genome sequences using the MAFFT program (Katoh et al., 2002). Nucleotide diversity (Pi) and total number of mutations (Eta) of plastid genome sequences were conducted through a sliding window with a window length of 600 bp and step size of 200 bp using DnaSP 6.10.03 (Rozas et al., 2017).

### Phylogenetic analysis

Phylogenetic analyses were performed with 47 species from the “core Lauraceae” group (Appendix S1), and two species of *Endiandra* R.Br. were treated as outgroup based on previous studies (Rohwer & Rudolph, 2005; Song et al., 2017b). After sequences were aligned by MAFFT 7.395 with auto strategy (Katoh et al., 2002), all 49 complete plastid genome sequences were modified manually (SSRs and indel events regions may not have aligned) in BioEdit 7.2.5 (Hall, 1999). Maximum-likelihood (ML) phylogenetic relationships were conducted in RaxML 8.2.10 tool (Stamatakis, 2014) using the CIPRES Science Gateway web (Miller, Pfeiffer & Schwartz, 2010) using the substitution model (GTRGAMMA) determined from jModelTest 2.1.9 (Darriba et al., 2012). Individual branches were assessed using the nonparametric bootstrap (BS) with 1,000 fast BS search performed in each analysis to obtain confidence support. Bayesian inference (BI) was performed with BEAST 1.8.3 for 50,000,000 generations using default parameters (Drummond et al., 2012). The best nucleotide substitution model, GTR+I+G, was estimated from jModelTest 2.1.9 (Darriba et al., 2012) using the Akaike information criterion methods. Uncorrelated relaxed clocks (Drummond et al., 2006) and the Yule process (Gernhard, 2008) were used in phylogenetic reconstruction. All parameter effective sample sizes were more than 200 according to Tracer v.1.6 (Rambaut, Drummond & Suchard, 2013), with the first 25% of sampled generations discarded as burn-in. Results of all phylogenetic analysis are displayed at Figtree 1.4.3 (http://tree.bio.ed.ac.uk/publications/).

## Results

### Complete plastid genome sequences

**Figure 1:**
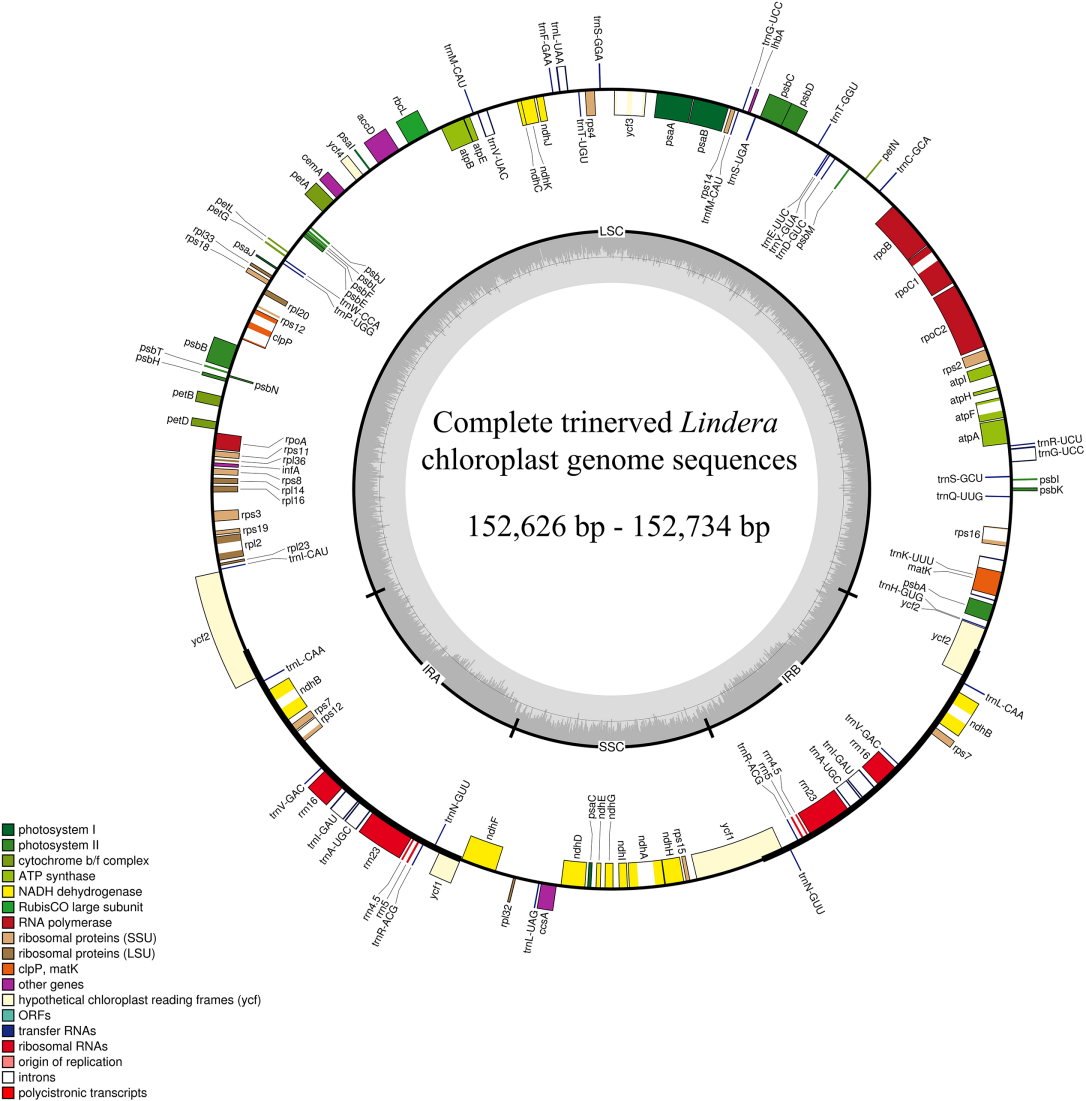
The plastid genomes map of 10 *Lindera* species. Genes shown inside the circle are transcribed clockwise, and those outside the circle are transcribed counter clockwise.

**Figure 2:**
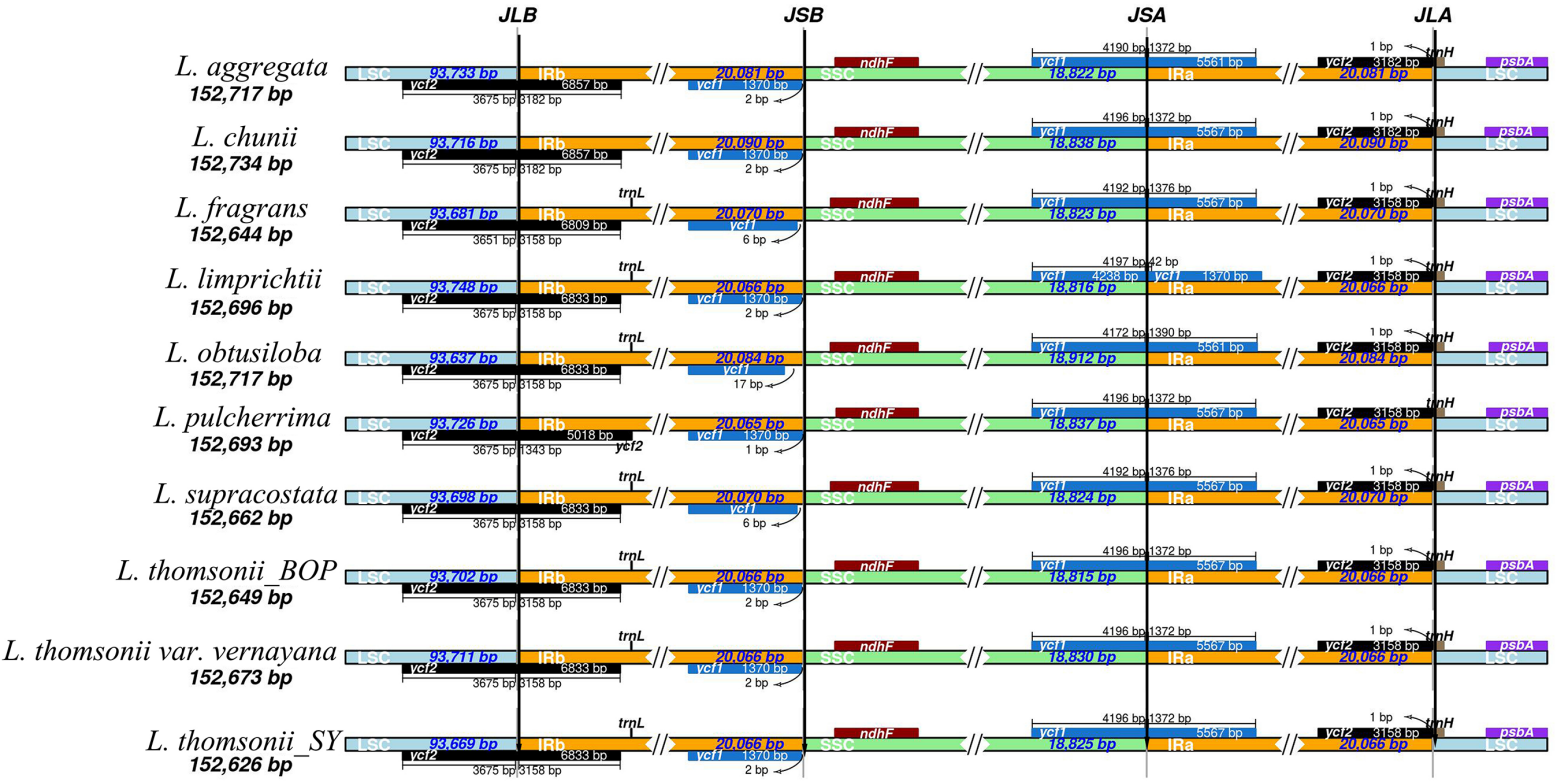
Comparison of the borders of the LSC, SSC, and IR regions among 10 *Lindera* plastid genome sequences. LSC, large single-copy; SSC, small single-copy; IR, inverted repeat.

After sequencing the plastid genome, we obtained more than six Gb of raw data from each *Lindera* species. The assembled plastid genome of the 10 *Lindera* species, with sizes ranging from 152,626 bp to 152,734 bp, was within the range of the other Lauraceae species (Song et al., 2017b). The total length of the *Lindera obtusiloba* plastid genome sequence was only 17 bp shorter than that of *Lindera chunii*, but it was longer than the other species (Table 1). All 10 *Lindera* plastid genomes showed a typical quadripartite circular structure (Fig. 1), consisting of a pair of inverted repeat (IR) regions divided by a small single copy (SSC) region and a large single copy (LSC) region. The entire *ycf1* crossed the SSC/IRb boundary, while 1,370 bp of 3′-*ycf1* were truncated at the boundary of the SSC/IRa. The entire *ycf2* crossed the LSC/IRa boundary, while the region 3,161 to 3,165 bp of 5′-*ycf2* was truncated at the boundary of the LSC/IRb (Fig. 2). The general G+C content of the full plastid genome was in the range of 39.1% to 39.2%, and the G+C content was 38%, 33.9%, and 44.4% in LSC, SSC, and IR regions, respectively. There was a total of 127 functional genes in the whole plastid genomes, including 83 protein coding genes, 36 tRNA genes, and eight rRNA genes (Table 1). Additionally, among these 10 plastid genomic sequences, 13 genes possessed only a single intron, and two genes (*ycf3* and *clpP*) possessed two introns.

### Number and forms of microstructural mutations

**Figure 3:**
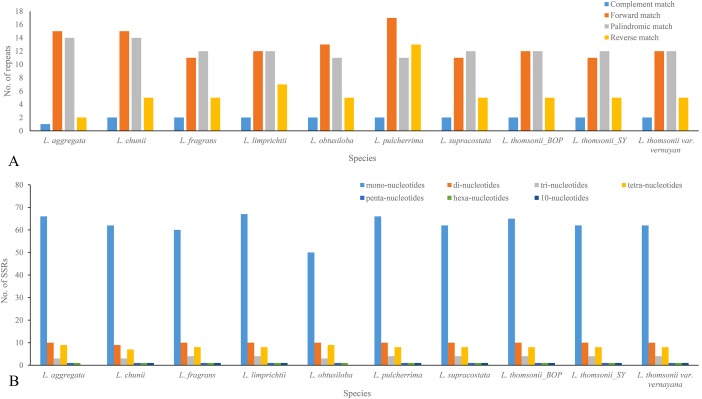
Repeats (A) and SSRs (B) in 10 *Lindera* species plastid genomic sequences. (A) Frequency of forward, palindrome, reverse, complement type repeats in the *Lindera* plastome sequences. (B) Number of SSRs in the *Lindera* plastome sequences.

When being tested as potential mutation sites, 30–43 repeats and 74–92 SSRs were located in the 10 plastome sequences, 31 repeats and 74 SSRs in the plastome of *Lindera obtusiloba*, 33 repeats and 92 SSRs in the plastome of *Lindera limprichtii*, and 43 repeats and 91 SSRs were in the plastome of *Lindera pilcherrima*, with other species within those ranges (Fig. 3A; Appendix S2). Most repeats were forward and palindromic matches, except *Lindera pulcherrima* had 13 repeats that were reverse matches. In 67.57% of *Lindera obtusiloba* and 73.81% of *Lindera chunii*, the majority of SSR motifs were mononucleotides. Two adjacent repeat units with mononucleotides A/G were in the *psbM/trnD*-GUC, and dinucleotide TC and mononucleotide T were in *ndhC/trnV*-UAC among all species. Nevertheless, eight *Lindera* species contained 10-nucleotide SSR within *trnL*-UAA, except for *Lindera aggregata* and *Lindera obtusiloba* (Appendix S3). In these SSR sites, we found 89 different ones among the four plastomes. There were 79 single nucleotide repeats of A/T ranging from 4 to 16 bp, seven single nucleotide repeats of G/C ranging from 8 to 27 bp (Fig. 3B), two 10-nucleotide repeats in the *atpH-atpI* and *trnT- trnL* gene spacer regions, and one 18-nucleotide repeat in the *trnF-ndhJ* gene spacer region (Appendix S3). Besides these SSR indels, 48 non-SSR indels were identified in these plastomes. The size of most non-SSR indels ranged from 1 to 26 bp, while that of the *psbM-trnD* gene spacer sequence was 41 bp (Appendix S3). Additionally, 20 motifs, including seven forward, 10 palindromic, two reverse, and one complementary repeat, were shared by all plastomes (Appendix S2). In addition, we manually identified six micro-inversion events in the regions of *rpl16* gene, *ccs-ndhD*, *petA-psbJ*, *rps3-rps19*, and *trnH-psbA* (Appendix S3). Two micro-inversions with three and eight bp were detected in the *trnH-psbA* region (Appendix S3). In the plastomes of all trinerved *Lindera* species, we detected 1,836 mutation events, including 156 indels and 1,674 substitutions, of which there were 24 double varieties, 181 parsimony informative sites, and 778 SNPs. Six hundred and eighty-four variable sites were in the LSC region, 226 in the SSC region, and 73 in the IR regions. There were 170 SNPs, including 591 transition (Ts) and 368 transversion (Tv) events in the plastomes.

### Genome sequence mutations and divergence

**Figure 4:**
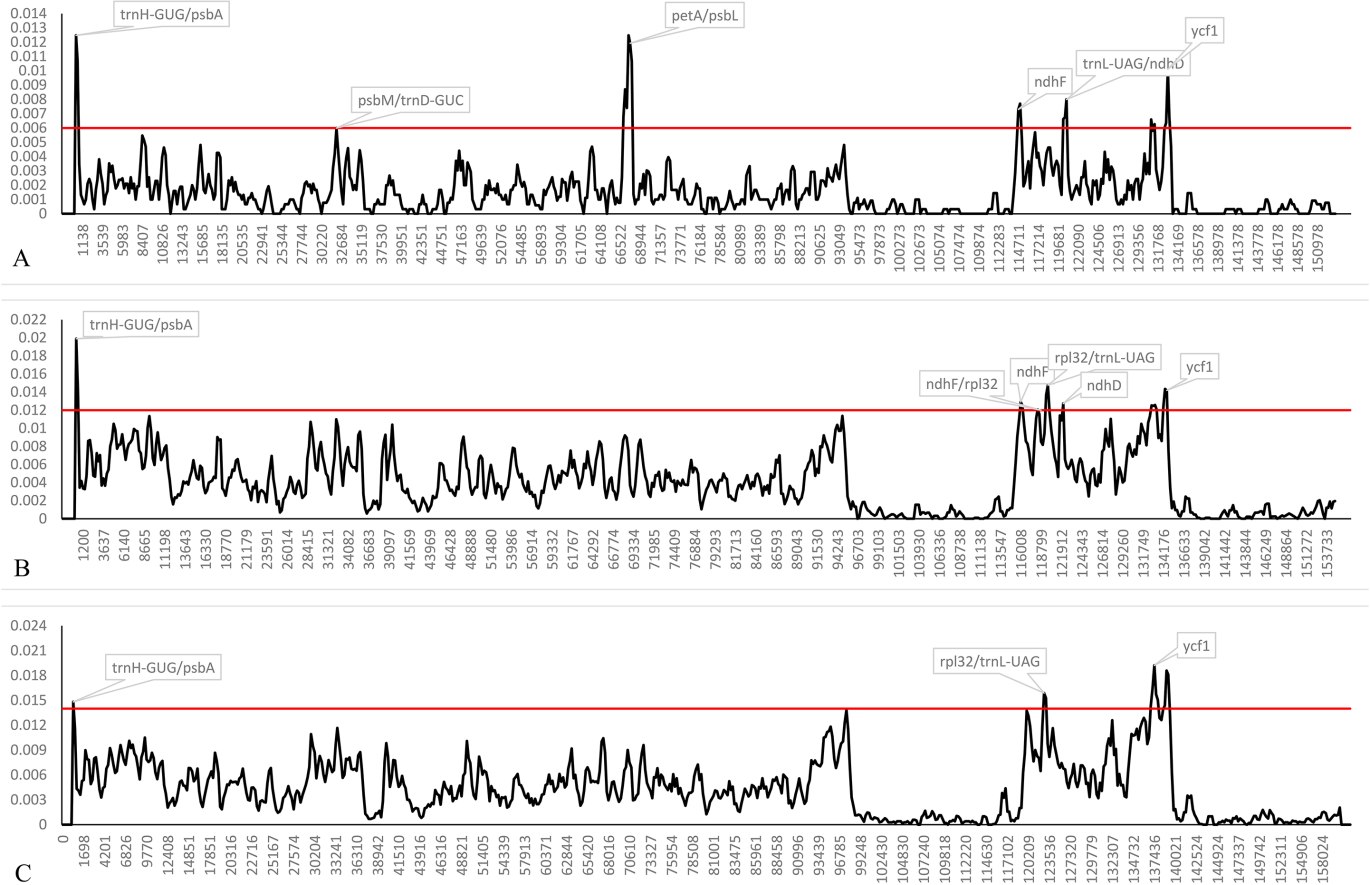
Sliding window analysis of the complete plastid genome sequences of trinerved *Lindera* species (A), 23 “core Laureae” species (B) and 47 “Core Lauraceae” species (C). Note: Window length: 600 bp; step size: 200 bp. Y-axis means nucleotide diversity.

Both indel and SNP markers were not randomly distributed in the plastomes, and we calculated the genetic divergence among plastomes of the four trinerved *Lindera* and all of the “core Laureae” species. The nucleotide diversity (Pi) values within 600 bp among the trinerved *Lindera* varied from 0 to 0.01248 with a mean of 0.00154, while among the 23 plastomes of the “core Laureae” species the values varied from 0 to 0.01989 with a mean of 0.00418 (Fig. 4A). Six highly variable loci (Pi >0.006) were identified in the plastomes of the trinerved *Lindera*. They were *trnH*-GUG/*psbA*, *psbM/trnD-*GUC, *petA/psbJ*, *ndhF, trnL-*UAG*/ndhD*, and *ycf1*. Different hypervariable regions including *ndhD, rpl32*/*trnL*-UAG, and three fragments of *ycf1* were precisely located in the plastomes of the “core Laureae” species (Fig. 4B). However, only five divergence hotspots were detected in the “core Lauraceae” plastome sequences of 41 species within the Persea group and Laureae-Cinnamomeae group, with values ranging from 0 to 0.01922 with a mean of 0.00447 (Fig. 4C), suggesting that two fragments of *ycf2*, *ndhD*, *ndhF*/*rpl32*, *ndhH*, and three fragments of *ycf1* may serve as useful DNA barcodes for Lauraceae. Therefore, these regions may be suitable prospective markers to reconstruct the phylogenetic relationship within *Lindera*, or the intergeneric relationship of Lauraceae.

### Phylogenetic analysis

**Figure 5:**
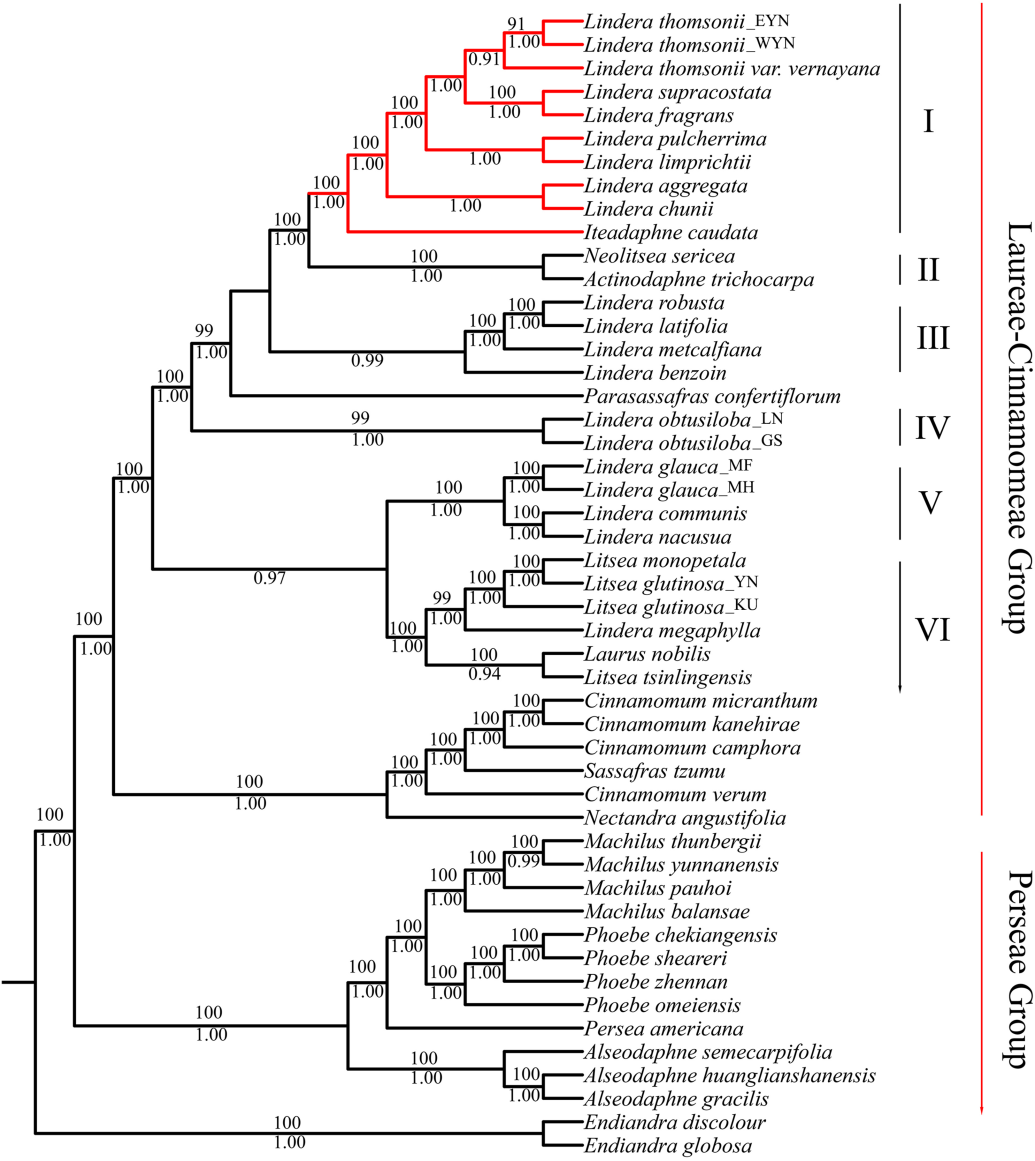
Complete plastid genome phylogenetic tree of 49 species based on maximum likelihood analysis and Bayesian inference. Branches with red color means trinerved *Lindera* species in this study. Numbers above branches are posterior probabilities and bellow branches are bootstrap values. EYN means East of Yunnan; WYN means West od Yunnan; LN means Liaoning; GS means Gansu; YN means Yunnan; MF, MH and KU mean sequence from Genbank.

The matrix of complete plastomes was used to reconstruct a phylogenetic tree of the 47 species from the “core Lauraceae” group. The topologies using ML and BI methods were similar. All species were divided into three groups: the Laureae group, the Cinnamomeae group, and the Perseae group. The Laureae group taxa were divided into six clades based on node support (Fig. 5). The posterior probability (PP) of BI analysis at most of the nodes was more than 0.90, and the BS values of the nodes were more than 90 in ML inference. Clade I contained the trinerved *Lindera* species, and *Iteadaphne caudata* with BS 100 and PP of 1.00. Clade II contained *Actinodaphne trichocarpa* C.K. Allen and *Neolitsea sericea* (Blume) Koidz. species with BS 100 and PP 1.00. Clade III contained *Lindera benzoin*, *Lindera robusta*, *Lindera latifolia*****, and *Lindera metcalfiana* species with PP 0.99. Clade IV contained two *Lindera obtusiloba* species. Clade V contained *L. communis*, *Lindera glauca*, and *Lindera nacusua* with BS 100 and PP 1.00. Clade VI contained *Laurus nobilis*, three *Litse*a species, and *Lindera megaphylla* with BS 100 and PP 1.00 (Fig. 5). Among these clades, eight trinerved *Lindera* species formed a monophyletic lineage which nested with the trinerved *Iteadaphne caudata*, and *Actinodaphne trichocarpa* and *Neolitsea sericea* formed the sister clade with BS 100 and PP 1.00 (Clades I and II). Clade III was supportive in a sister position to Clades I and II in ML and BI trees.

## Discussion

In this study, we accomplished 10 complete plastid genomes of trinerved *Lindera* species using Illumina high-throughput sequencing technology (Fig. 1). The sizes of the trinerved *Lindera* plastomes were very similar to each other—longer than *Lindera benzoin* (152,478 bp) and *Lindera nacusua* (152,211 bp), but smaller than *Lindera metcalfiana* (152,968 bp) and *Lindera robusta* (152,852 bp) (Zhao et al., 2018). All species shared a typical angiosperm quadripartite structure, and the IR regions were more conservative than the LSC and SSC regions (Figs. 2 and 4; Appendix S4). Further analysis on sequence conservation between the 10 trinerved *Lindera* species and 18 previously sequenced “core Laureae” species has been carried out by using the BLAST tool, with results showing high similarities in both conserved structure and gene content (≥99%) (Chen et al., 2017; Li et al., 2017; Song et al., 2015, 2016, 2017b, 2018; Wu et al., 2017; Wu, Ho & Chang, 2016). In addition, the nucleotide variable value of the whole plastomes among the trinerved *Lindera* species was 0.14%. This value is approximate to the value of two *Machilus* species (0.15%) (Song et al., 2015), two *Phoebe* species (0.10%) (Song et al., 2017a), and three *Alseodaphone* species (0.12%) (Song et al., 2018), which indicates that the genetic divergence within the genera in the family Lauraceae is low (Appendix S4).

Microstructural mutations including perfect SSRs with a minimum size of 10 bp were detected in the plastid genomes of the trinerved *Lindera* species. Similarly, 83, 88, 81, 82, 86, 65, and 66 SSRs were recognized among the plastid genomes of *Cinnamomum camphora* (L.) T. Nees & C.H. Eberm, *Cinnamomum micranthum* Hayata, *Litsea glutinosa*, *Machilus yunnanensis* Lecomte, *Persea americana* Mill., *Phoebe bournei* (Hemsl.) Yang, and *Phoebe chekiangensis* P.T. Li, respectively (Chen et al., 2017; Li et al., 2017). Among the trinerved *Lindera* plastomes, mononucleotide repeats (A/T) were found to be abundant among SSRs (about 68–74%), followed by dinucleotide repeats (AT/AT and AG/CT, 10–13%) among the plastome sequences (Fig. 3B). This suggested that mononucleotide repeats may contribute more phylogenetic signal information in comparison to other SSRs (Appendix S3). Also, repeat motifs are generally found in the intergenic regions of trinerved *Lindera* plastid genomes, which shows the great variable nucleotide regions in the plastid genomes (Appendices S2 and S3). Repeat numbers were similar to *Cinnamomum camphora*, *Cinnamomum micranthum*, *Litsea glutinosa*, *Persea americana*, *Phoebe bournei*, and *Phoebe chekiangensis*, abundant palindromic repeats (Chen et al., 2017; Li et al., 2017; Song et al., 2016; Wu et al., 2017; Wu, Ho & Chang, 2016), which may provide some secondary structures or duplicate sequences in the whole genome (Kurtz et al., 2001).

All phylogenetic trees supported the relationships among the “core Lauraceae” described by Rohwer & Rudolph (2005) as a sister group of the Laureae-Cinnamomeae group and *Persea* group in accordance with other previous studies (Chanderbali, van der Werff & Renner, 2001; Huang et al., 2016; Li et al., 2011; Rohwer, 2000; Rohwer et al., 2009; Rohwer & Rudolph, 2005; Song et al., 2017b). The BI and ML analyses with complete plastid genomes yielded 1.00 Bayesian PPs and 100 BS values at each node. The relationships among the 20 *Lindera* species and 13 representatives of eight other genera species in the Laureae-Cinnamomeae group were phylogenetic trees divided into six clades, as described by Rohwer & Rudolph (2005). All trinerved *Lindera* species belonging to sect. *Daphnidium* were monophyletic. The phylogenetic trees also showed that the trinerved *Lindera* species had a sister relationship with *Iteadaphne caudata*, and they grouped with *Neolitsea* and *Actinodaphne* with higher variables among *ndhF*, *rpl32*/*trnL*-UAG, and *ycf1*, similarly to how Fijridiyanto & Murakami (2009) constructed phylogenetics with a low copy nuclear *rpb2* gene, and how Song et al. (2017b, 2019) did the same with plastid genomes. Consequently, the systematics of *Iteadaphne* were recombined from *Lindera caudata* (Nees) Hook. f. by a single floret per umbellule, while the type specimen of *Parasassafras*, based on *Actinodaphne confertiflora* Meisn., was appropriated (Li, 1985; Ng, 2005; Tsui, 1987a, 1987b). Our results support that the number of anther cells is not a valuable characteristic, and the hypothesis of *Neolitsea* and *Actinodaphne* being more closely related to the trinerved *Lindera* species and *Iteadaphne* needs further studies based on intensive sampling.

## Conclusions

Two highly variable regions, *ndhF* and *psbA*-*trnH*, were identified among the trinerved *Lindera* species, and their highly conserved structures and gene content have been found to be very similar. This similarity may be useful when studying phylogenetic information and in species identification. Our results also supported that the sect. *Daphnidium* species, as an independent sub-clade with *Iteadaphne caudata*, formed a sister clade to *Actinodaphne* and *Neolitsea*. The results also show that the systematics of *Iteadaphne* were recombined from *Lindera caudata* (Nees) Hook. f. when a single floret per umbellule was misappropriated, which indicated a close relationship with the trinerved *Lindera* species. Altogether, this study will help improve our understanding of phylogenetics and the evolution of the *Lindera* species.

## Supplemental Information

10.7717/peerj.7662/supp-1 Supplemental Information 1 Raw data of 10 *Lindera* species. Complete plastid genome sequences and annotations of *Lindera aggregata*, *Lindera chunii*, *Lindera limprichtii*, *Lindera pulcherrima*, *Lindera obtusiloba*, *Lindera supracostata*, *Lindera thomsonii* BOP, *Lindera thomsonii* SY, *Lindera thomsonii* var. vernayana, *Lindera fragrans*.

10.7717/peerj.7662/supp-2 Supplemental Information 2 Characteristic of 10 Lindera plastome sequences. Appendix S1 List and accession number of 49 taxa for complete plastid genome phylogenetic analysis
Appendix S2 The repeats distribution in the plastid genome sequences of Lindera species (Note: F, P, R, and C represent forward, palindrome, reverse, and complement type repeat sequences in the plastome.)
Appendix S3 The mutations and Indels distribution in the plastid genome sequences of Lindera species

10.7717/peerj.7662/supp-3 Supplemental Information 3 VISTA-based identity plots showed sequence identity of 10 Lindera plastid genome sequences with *Laurus nobilis* as a reference. Appendix S4 VISTA-based identity plots showed sequence identity of 10 Lindera plastid genome sequences with *Laurus nobilis* as a reference.

10.7717/peerj.7662/supp-4 Supplemental Information 4 Tree flies of 10 *Lindera* species. Appendix S5 tree file of Bayesian inference based on BEAST 2.4.8. Appendix S6 tree file of maximum likelihood based on RAxML 8.2.10.

10.7717/peerj.7662/supp-5 Supplemental Information 5 GenBank and LCGD numbers

10.7717/peerj.7662/supp-6 Supplemental Information 6 Sequence data

## Additional Information and Declarations

### DNA Deposition

The following information was supplied regarding the deposition of DNA sequences: Sequence numbers can be found in the Supplemental File.

### Data Availability

The following information was supplied regarding data availability: The raw measurements are available in the Supplemental Files.
