# Supplementary material for: Plastome sequences help to improve the systematic position of trinerved Lindera species in the family Lauraceae
Source: PeerJ. 2019 Oct 7;7:e7662. doi: 10.7717/peerj.7662 (PMC6786250; doi:10.7717/peerj.7662)

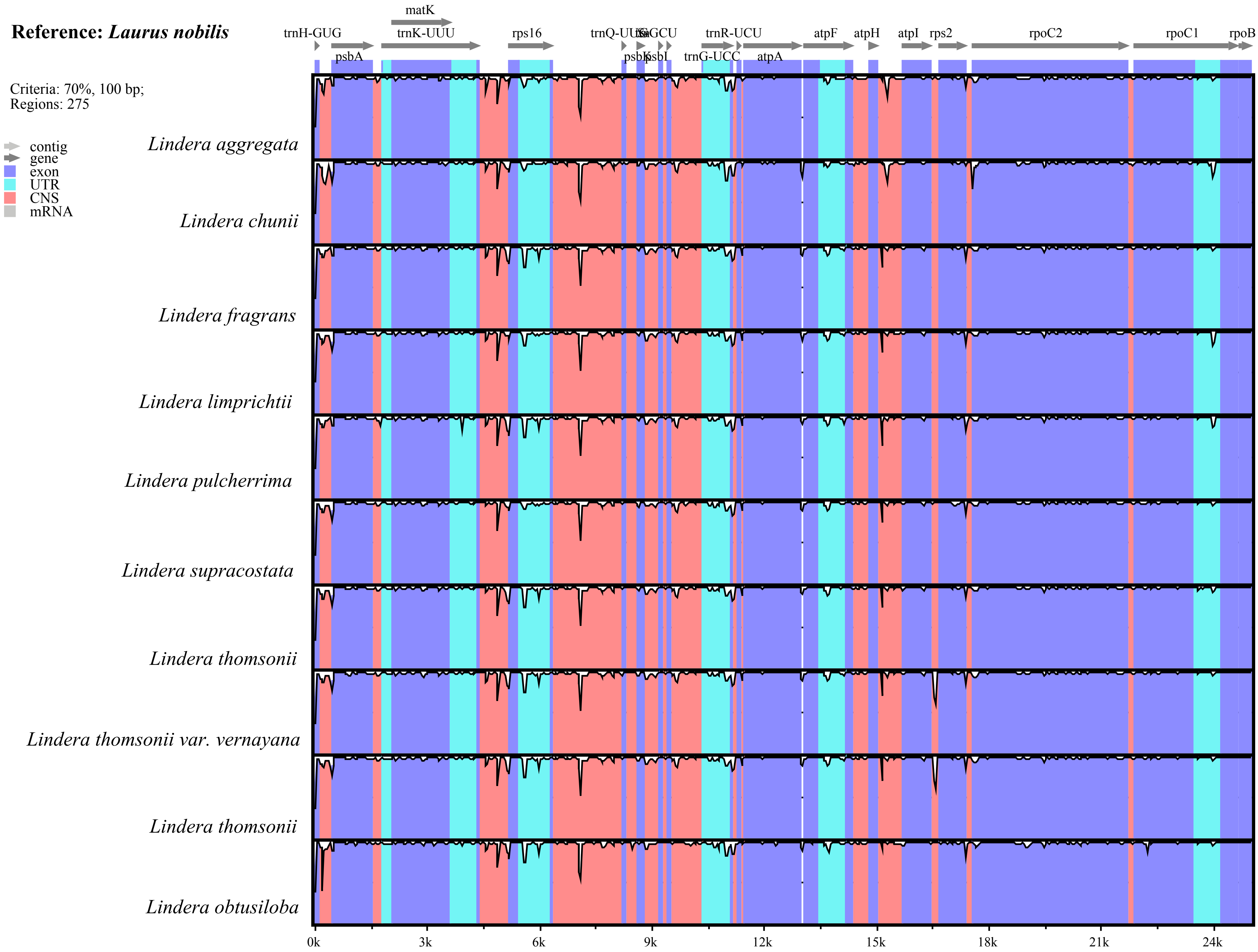

Fig S4. VISTA-based identity plots showed sequence identity of *Lindera* sequenced chloroplast genomes with *Laurus nobilis* as a reference.

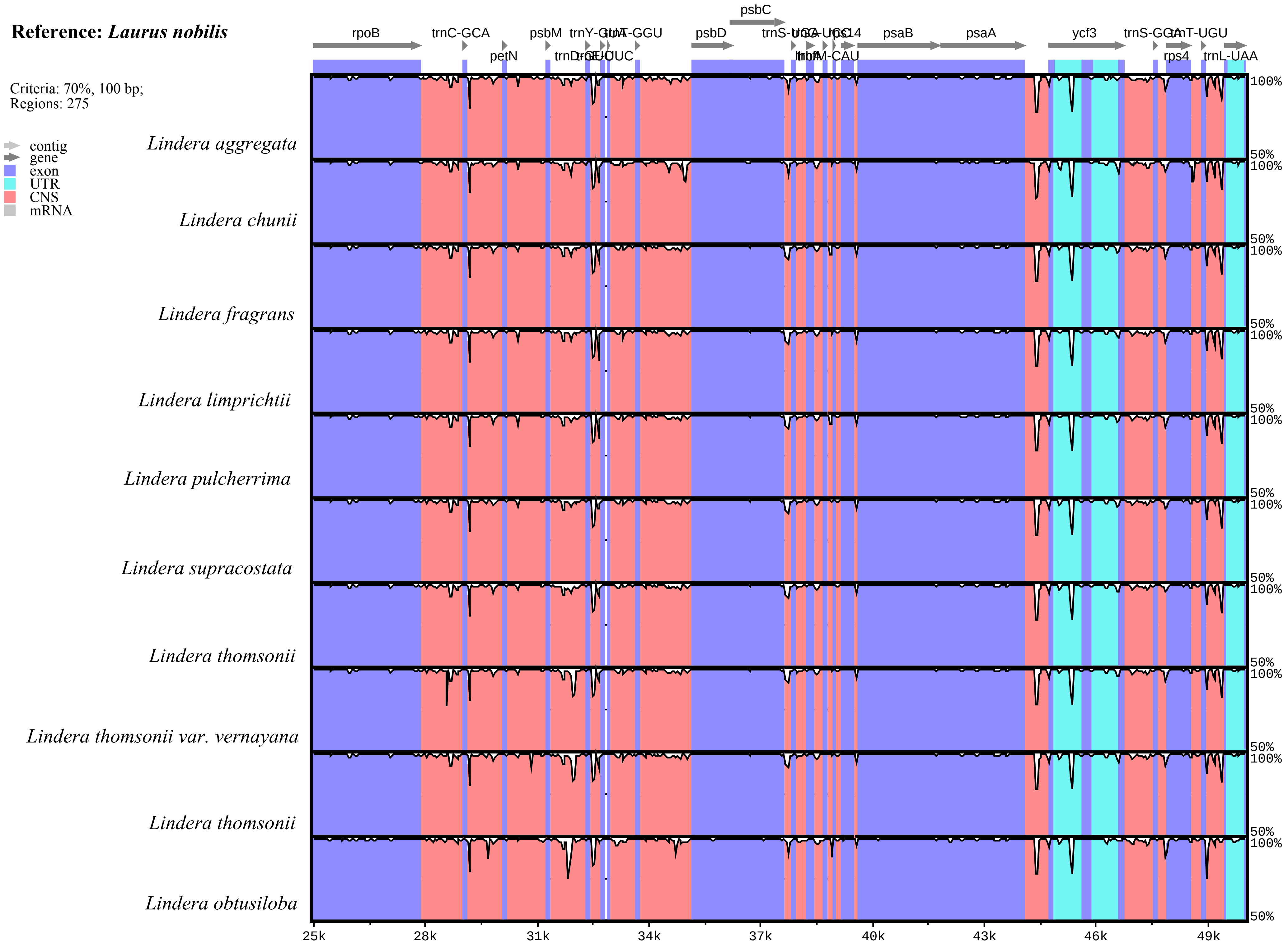

Reference: Laurus nobilis
Criteria: 70%, 100 bp;
Regions: 275
contig
gene
exon
UTR
CNS
mRNA
rpoB
trnC-GCA
petN
psbM
trnY-GUA
trnD-GUC
trnE-UUC
trnT-GGU
psbD
psbC
trnS-UGA
trnG-UCC
trnfM-CAU
rps14
psaB
psaA
ycf3
trnS-GGA
rps4
trnT-UGU
trnL-UAA
Lindera aggregata
Lindera chunii
Lindera fragrans
Lindera limprichtii
Lindera pulcherrima
Lindera supracostata
Lindera thomsonii
Lindera thomsonii var. vernayana
Lindera thomsonii
Lindera obtusiloba
100%
50%
25k
28k
31k
34k
37k
40k
43k
46k
49k

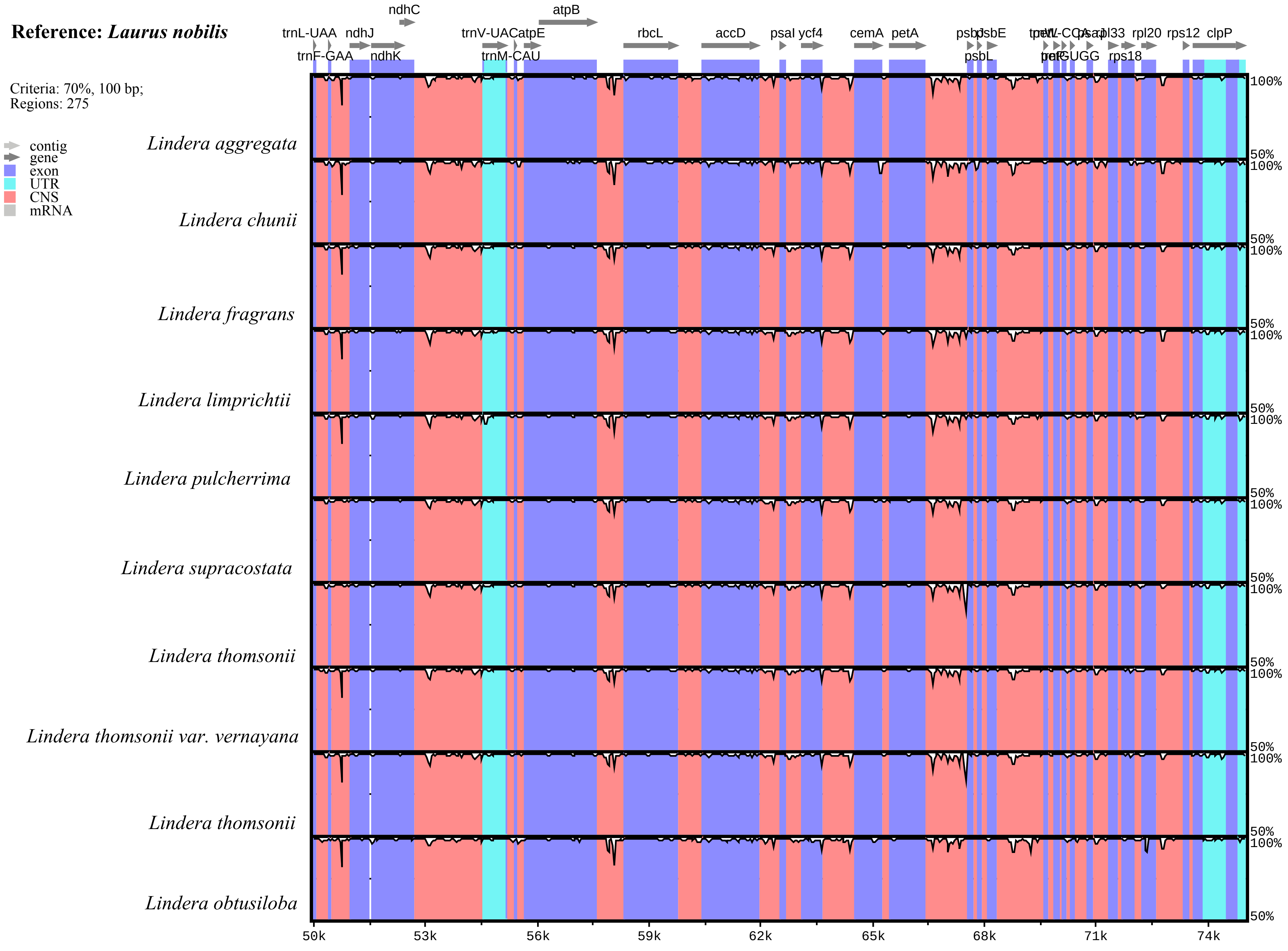

Reference: Laurus nobilis
Criteria: 70%, 100 bp;
Regions: 275
contig
gene
exon
UTR
CNS
mRNA
ndhC
atpB
trnL-UAA
ndhJ
trnV-UAC
atpE
rbcL
accD
psaI
ycf4
cemA
petA
psbE
rpl20
rps12
clpP
trnF-GAA
ndhK
trnM-CAU
psbL
rps18
Lindera aggregata
Lindera chunii
Lindera fragrans
Lindera limprichtii
Lindera pulcherrima
Lindera supracostata
Lindera thomsonii
Lindera thomsonii var. vernayana
Lindera thomsonii
Lindera obtusiloba
100%
50%
50k
53k
56k
59k
62k
65k
68k
71k
74k

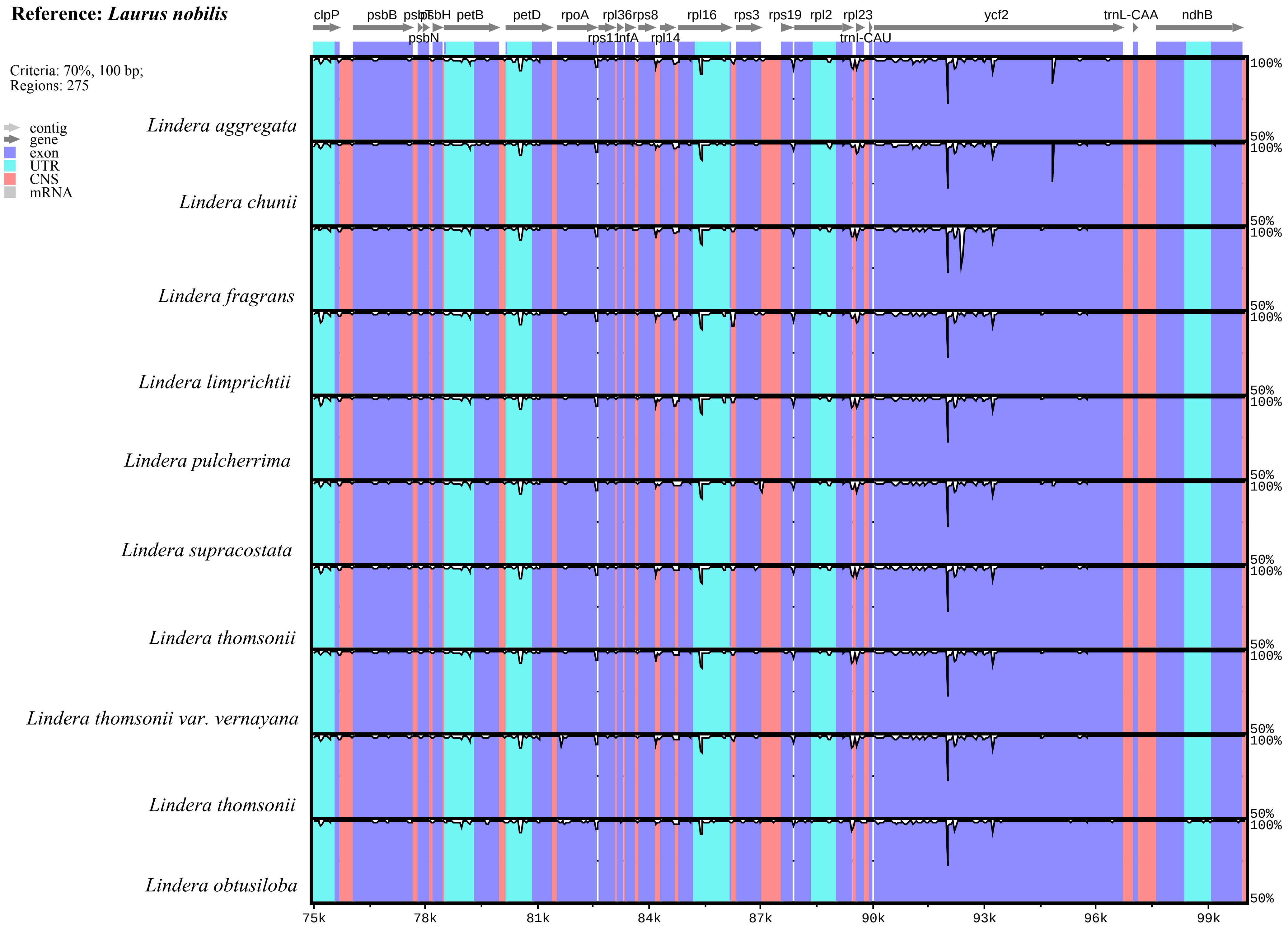
Reference: Laurus nobilis
Criteria: 70%, 100 bp;
Regions: 275
contig
gene
exon
UTR
CNS
mRNA
clpP
psbB
psbT
psbH
petB
psbN
petD
rpoA
rpl36
rps8
rps11
infA
rpl14
rpl16
rps3
rps19
rpl2
rpl23
trnI-CAU
ycf2
trnL-CAA
ndhB
Lindera aggregata
Lindera chunii
Lindera fragrans
Lindera limprichtii
Lindera pulcherrima
Lindera supracostata
Lindera thomsonii
Lindera thomsonii var. vernayana
Lindera thomsonii
Lindera obtusiloba
100%
50%
75k
78k
81k
84k
87k
90k
93k
96k
99k

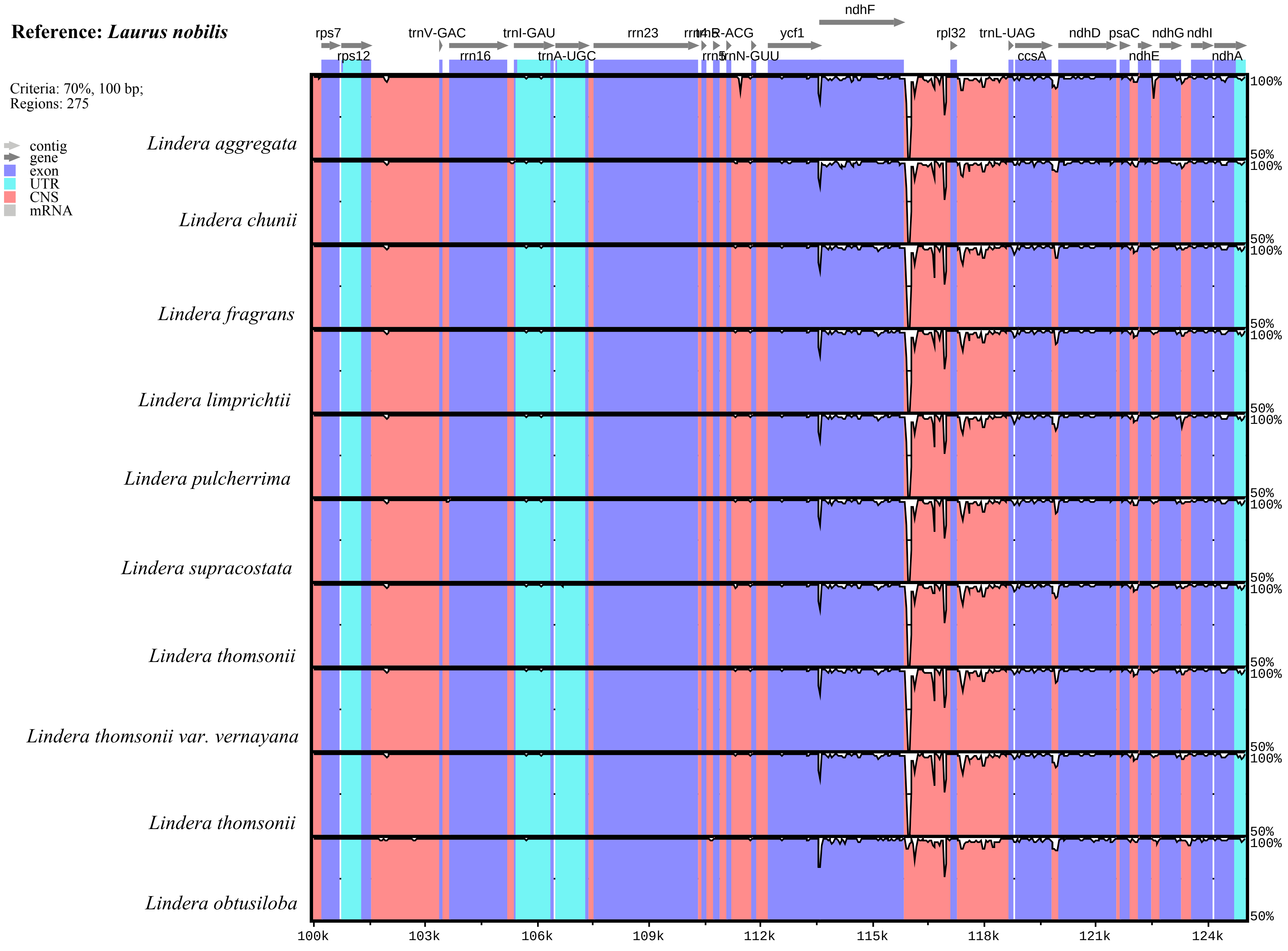

Reference: Laurus nobilis
Criteria: 70%, 100 bp;
Regions: 275
contig
gene
exon
UTR
CNS
mRNA
Lindera aggregata
Lindera chunii
Lindera fragrans
Lindera limprichtii
Lindera pulcherrima
Lindera supracostata
Lindera thomsonii
Lindera thomsonii var. vernayana
Lindera thomsonii
Lindera obtusiloba
rps7
rps12
trnV-GAC
rrn16
trnI-GAU
trnA-UGC
rrn23
trnR-ACG
rrn5
trnN-GUU
ycf1
ndhF
rpl32
trnL-UAG
ccsA
ndhD
psaC
ndhE
ndhG
ndhI
ndhA
100%
50%
100k
103k
106k
109k
112k
115k
118k
121k
124k

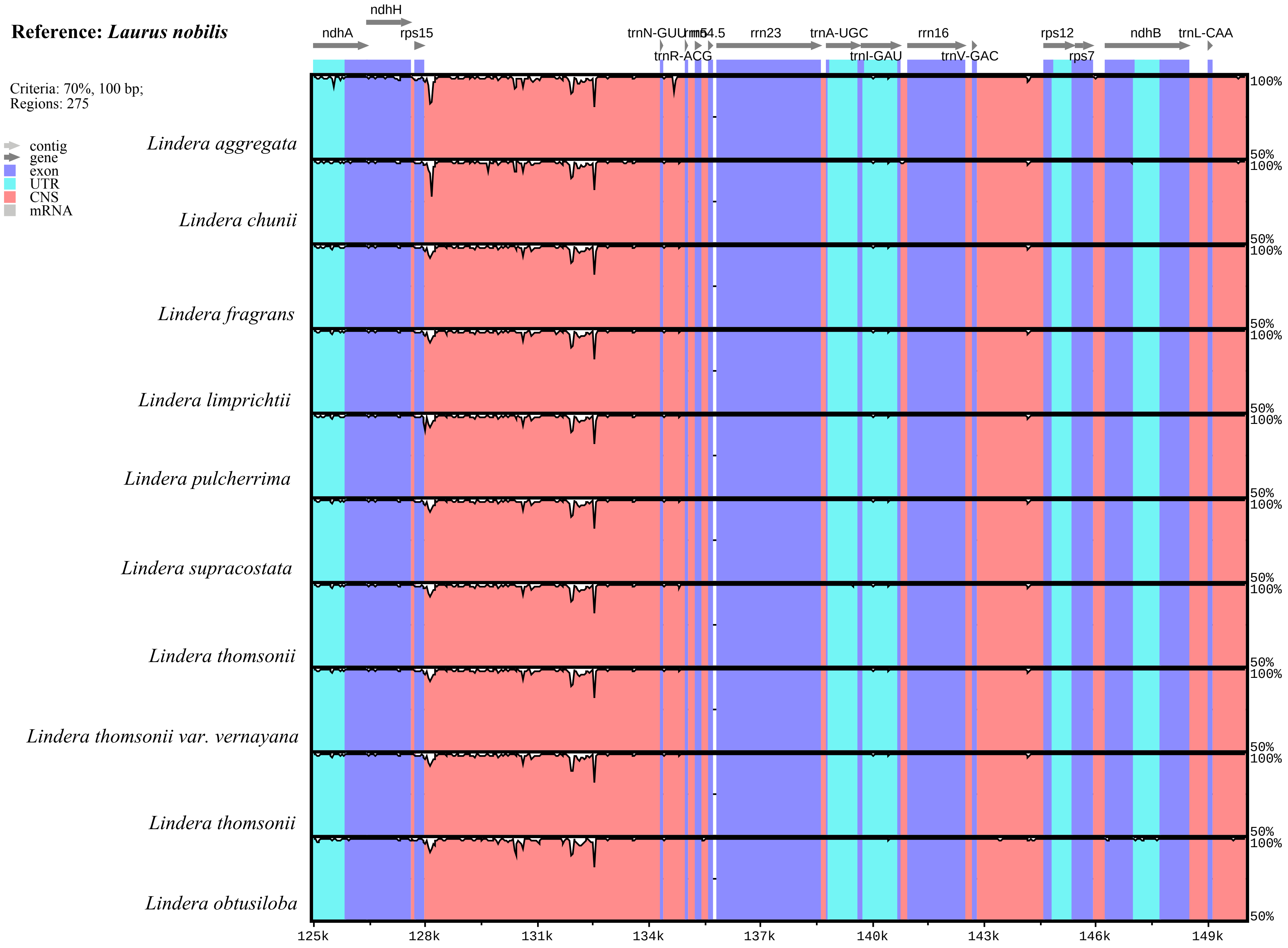

Reference: Laurus nobilis
Criteria: 70%, 100 bp;
Regions: 275
contig
gene
exon
UTR
CNS
mRNA
ndhH
ndhA
rps15
trnN-GUU
rrn5
rrn4.5
rrn23
trnA-UGC
rrn16
rps12
ndhB
trnL-CAA
trnR-ACG
trnI-GAU
trnV-GAC
rps7
Lindera aggregata
Lindera chunii
Lindera fragrans
Lindera limprichtii
Lindera pulcherrima
Lindera supracostata
Lindera thomsonii
Lindera thomsonii var. vernayana
Lindera thomsonii
Lindera obtusiloba
100%
50%
125k
128k
131k
134k
137k
140k
143k
146k
149k

Reference: ***Laurus nobilis***

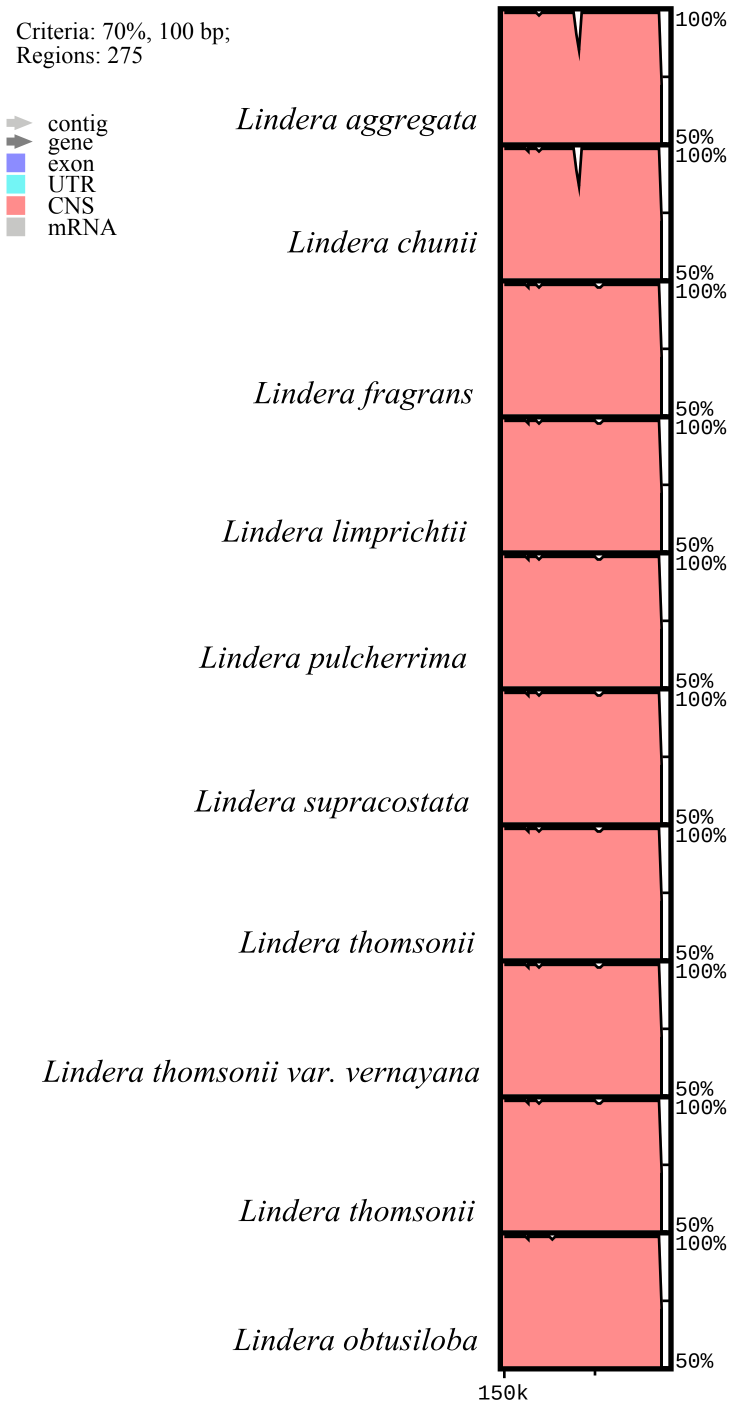

Supplement: Supplemental Information 3 — Appendix S4 VISTA-based identity plots showed sequence identity of 10 Lindera plastid genome sequences with Laurus nobilis as a reference. [file peerj-07-7662-s003.pdf]
